# Cognitive, Motor and Social Factors of Music Instrument Training Programs for Older Adults’ Improved Wellbeing

**DOI:** 10.3389/fpsyg.2019.02868

**Published:** 2020-01-10

**Authors:** Jennifer MacRitchie, Matthew Breaden, Andrew J. Milne, Sarah McIntyre

**Affiliations:** ^1^The MARCS Institute for Brain, Behaviour and Development, Western Sydney University, Penrith, NSW, Australia; ^2^Center for Social and Affective Neuroscience, Linköping University, Linköping, Sweden

**Keywords:** older adults, music learning, fine motor skills, cognitive training, health, wellbeing

## Abstract

Given emerging evidence that learning to play a musical instrument may lead to a number of cognitive benefits for older adults, it is important to clarify how these training programs can be delivered optimally and meaningfully. The effective acquisition of musical and domain-general skills by later-life learners may be influenced by social, cultural and individual factors within the learning environment. The current study examines the effects of a 10-week piano training program on healthy older adult novices’ cognitive and motor skills, in comparison to an inactive waitlisted control group. Fifteen participants completed piano training led by a music facilitator in small groups (max *n* = 4 per lesson class; two experimental, two waitlisted control groups). Data was collected using an explanatory sequential design: quantitative data from a battery of cognitive and motor tests was collected pre/post-test on all participants, with further post-test data from the waitlisted control group (*n* = 7). Qualitative data included weekly facilitator observations, participant practice diaries, and an individual, semi-structured, post-experiment interview. Bayesian modelling demonstrated moderate evidence of a strong positive impact of training on part A of the Trail Making test (TMT), indicating improved visuo-motor skills. Moderate evidence for negative impacts of training on part B of the Trail Making Test (and difference score delta) was also found, suggesting no benefit of cognitive switching. Qualitative results revealed that the group learning environment motivated participants to play in musical ensembles and to socialize. Motivation was optimal when all participants were happy with the chosen repertoire (participants reported they were motivated by learning to play familiar music) and when the facilitator observed that groups had formed cohesive bonds. Informed by these factors, exploratory analyses demonstrated strong evidence that a participant’s lesson class had an impact on post-test scores (TMT part A). These results not only demonstrate the extent of cognitive benefits of a short-term piano training intervention for older adults, but also the importance of considering the group dynamics in the learning environment.

## Introduction

There is increasing recognition that participation in music has the potential to benefit an individual’s health and wellbeing, but the full scope of these benefits and the best activities for optimizing outcomes are unknown (Krause et al., 2018). As the worldwide population is aging, it is important to explore the capacity for non-pharmacological interventions to stave off age-related declines. In order to understand the precise mechanisms by which arts engagement practices can help older adults to maintain physical and mental skills, we need hypothesis-driven, intervention-based research that incorporates quantitative measures to target specific motor and cognitive outcomes. At the same time, exploratory and qualitative methods can help to determine how these artistic activities can be delivered in a meaningful and practical way, while optimizing participants’ wellbeing gains. In the current study, we combined these approaches in investigating the benefits of learning piano for older adults.

Older adults experience a myriad of psychosocial benefits from learning to play a musical instrument, even beginning as novices and training over relatively short-term periods (Jutras, 2006; Bugos et al., 2007; Taylor and Hallam, 2008; Seinfeld et al., 2013; Varvarigou et al., 2013; Creech et al., 2014; Roulston et al., 2015; Bugos et al., 2016; Bugos and Kochar, 2017). A recent scoping review of eleven studies found a correlation between music playing and cognitive benefits for older adults (Schneider et al., 2018). As the majority of these studies are correlational and consider a broad range of different types of music activities, this means that the causal relationship between music instruction and wellbeing benefits for older adults is still undetermined. It is possible that it is not musical activity that causes these benefits: an alternate explanation may be that older people who choose to engage in musical activity also happen to have higher function. For this reason, we used an intervention-based design, assigning participants randomly into either an experimental group that received music training, or to a control group.

There are four existing intervention studies for healthy older adults in the literature that utilize a training program on a musical instrument in conjunction with, or instead of voice. Bugos and colleagues demonstrate that healthy older adults experience significant improvements in cognitive measures (particularly the Trail Making Test and Digit Span Test) as a result of piano training programs when compared against a control group (Bugos et al., 2007). Seinfeld and colleagues also show significant improvements in cognitive measures (one of these being the Trail Making Test part A) for a group of older adults involved in piano training programs as compared to other leisure activities (e.g., exercise or painting) (Seinfeld et al., 2013). A recent study using voice and percussion in rhythmic and improvization exercises (Biasutti and Mangiacotti, 2018) supports the hypothesis that older adults’ general cognitive skills are improved in comparison to those completing a non-musical control activity (exercise). Here, the experimental group showed significant improvements in the Mini-Mental State Examination, and a trend for improvements in the Trail Making Test Part A while the control group’s performance remained stable. A short-term mallet training program has also been shown to lead to significant increases in musical self-efficacy and a trend increase in performance of the Trail Making Test part B in comparison to an autobiographical discussion group (Bugos and Cooper, 2019). In our study, we also included the TMT, to test the hypothesis that piano training improves performance on this task.

Although gains in general cognition have been shown across these four intervention studies, music instrument learning may also be an effective task for improving fine motor skills. Sensorimotor function generally declines with age (Seidler et al., 2010), and performance of the upper limb in visuomotor tasks are also subject to this decrease (Lee et al., 2013). The tasks required in music instrument training, employing sensory, motor and multimodal brain regions, have been shown to stimulate brain plasticity (Zatorre et al., 2007; Altenmüller and Schlaug, 2015). This would suggest that auditory-motor training that is inherent in Western formal music instruction^1^ may be likely to improve an individual’s fine motor skill. Piano playing in particular trains both coupled movements across the fingers (used when manipulating objects with the hands) and individuated finger movements (independent finger use key to dexterity) (Furuya and Altenmüller, 2013). This is a contributing factor to the success of using piano training programs for rehabilitation [e.g., in stroke-affected hands (Villeneuve et al., 2014)], but it is unclear if this type of training is useful for maintenance or improvement in the context of aging. Preservation of domain-general fine motor skills may also benefit healthy older adults, supporting maintenance of skills required for numerous daily tasks involved in independent living. In the current study, we included tests of both cognitive and physical developments in older adults as a result of a formal Western facilitator-led music instrument training program.

Current research on the best practices for teaching music still has some way to go to fill the gap between promoting life-long learning in music, and the current research focus which is typically on child development, and sustaining engagement throughout adolescent years (Creech and Hallam, 2016; Krause and Davidson, 2018). It is clear that older participants in a variety of contexts gain cognitive, emotional and social benefits from developing the skills to play a musical instrument, taking place in a range of formal and informal learning environments (Veblen, 2012; Drummond, 2018). An increase in music-specific self-efficacy for older adults can occur even over relatively short time-frames (Bugos et al., 2016). Older adults frequently cite the ensemble nature of musical activities as a motivating factor to continuing engagement in learning to play an instrument (Roulston et al., 2015), with the social aspect of the ensemble offering its own wellbeing benefits through developing new relationships and decreasing isolation. With this in mind, it is important to clarify how these group music instrument lessons can be delivered meaningfully to take advantage of these features. As Andrea Creech, Susan Hallam and colleagues have noted in the Music for Life project (Creech et al., 2014), the effective acquisition of musical skills by later-life learners may be highly dependent on their subjective experiences in the dynamic learning environment, and the use of learning materials appropriate to their abilities and interests. Choice of repertoire, opportunities for peer interaction, and good use of aural/visual materials contributed to the learners’ satisfaction with the program. The authors noted the importance and influence of the facilitator (both their interpersonal and teaching skills) on the outcomes of older adults’ informal music learning (Creech and Hallam, 2016).

It is important to consider that older adults are not a homogenous group; in reality there is a mixture of abilities, preferences, and cultural backgrounds within this population (Creech et al., 2012). Given that older adults’ physical and mental declines are experienced across a wide spectrum that does not simply align to chronological age, it is entirely possible that a group of novice learners will comprise a mix of abilities. The field of adult education acknowledges older adults not only as a group potentially with reduced ability, but also as a group rich in knowledge, experience and motivation (Dabback and Smith, 2012).

As fine motor skills (coupled and individuated finger movements) are developed when learning to play the piano, our research question considered: to what extent are fine motor skills developed within a short (10-week) piano training program associated with improvements in domain-general fine motor skills for older adults? Our hypothesis was that the participants who had received training would show more improvements in general motor skills than those who had not received the training^2^. By collecting participants’ subjective experiences of the program, we also planned to examine the important elements of music training programs for older adult novices. School-age students’ motivations to continue engagement with music training have been shown to be related to fulfilling basic needs in competence, relatedness and autonomy (Evans et al., 2013). Through the paradigm of continuing learning and life-long learning, we can expect older adults to differ from young students in terms of their motivations and experiences (the learner), their independence and choice of how and where they learn (context), and their process of learning (Roulston, 2010). Older adults are generally expected to be more independent learners who are keen to be in control of their learning (self-directed). This self-directed learning is seen as a tenet of andragogy (the methods and principles behind older adult teaching and learning) but the extent to which this is evidenced in music learning appears to vary across groups of older adults in different musical contexts, e.g., in community ensemble rehearsals (Kruse and Texas, 2009). Differences in older adults’ motivations and degree of self-directed learning may also contribute to how a group learns together, with challenges arising from differing levels of expertise, and different ways of relating and belonging to a larger community (Kruse and Texas, 2009; Kruse, 2012). Older adults vary in terms of their expertise, their experience, and how they want to learn; each of these elements cannot be considered individually, as they exist in tandem with a wide variety of personality and social characteristics. The group dynamics are important as they are often key to sustaining motivation to learn (Veblen, 2012). Music educators themselves see a need to shift music training from a standpoint of expertise training to one involving different cultural and social aspects of music (Krause and Davidson, 2018). It is pertinent, therefore, to consider some of the social aspects of music lessons for older adults; how these might be important and how these might influence their acquisition of skill as well as their wellbeing outcomes. We did not manipulate the group cohesiveness, or personality types assigned to any one class in the current study. Instead, we performed *post hoc* comparisons between the various classes to examine whether group dynamics were related to individuals’ cognitive and motor achievements.

## Materials and Methods

### Design

The current study used an explanatory sequential design within an intervention design (Creswell, 2015): essentially, in the context of running an intervention study with pre/post data collection, quantitative data was collected at timepoints pre and post the intervention, and qualitative data was collected throughout and on completion of the intervention. This allowed us to capture participants’ subjective experiences of the program, as well as their progress in acquiring domain-specific instrument-playing skills and domain-general cognitive and fine motor skills.

**FIGURE 1:**
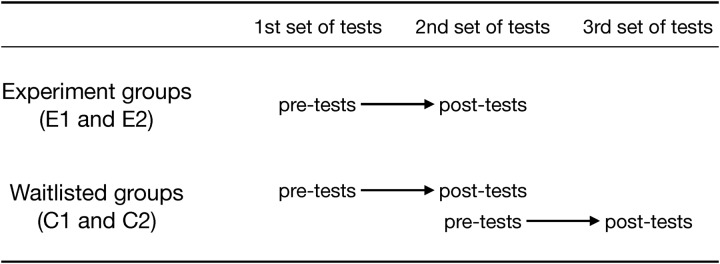
The structure of the experiment. All sets of tests are identical at each timepoint. Note that the second set of tests undertaken by the waitlisted control group serve as the post-test relative to their first set of tests; but they serve as the pre-test to their third set of tests (which are post-tests).

On recruitment, participants were allocated into either an active experimental group or a waitlisted inactive control group who received the music instrument training after the 2nd set of tests had been conducted. This control group submitted to a further round of post-tests once they too had completed the intervention. Test timing and group allocation can be seen in Figure 1.

### Participants

Participants were recruited in response to local newspaper and social media adverts (volunteer sampling). Seventeen older adult novices were recruited to the project (13F, 4M, age *M* = 70.9, *SD* = 5.5 years) under the following inclusion criteria: (i) aged 65 years or over, (ii) less than 2 years of formal music instrument training, (iii) no physical impairments to the hands or arms, (iv) no cognitive impairments, (v) normal to corrected hearing and vision. All participants were right-handed. Inclusion criteria (ii), (iii) and (v) were assessed via participant self-report. Before baseline testing, all participants were screened for cognitive impairments [inclusion criteria (iv)] using the short version of Addenbrooke’s Cognitive Examination III (M-ACE) (Hsieh et al., 2013) and were required to achieve a score of 25 or above.

A previous pilot study using a similar training program had revealed large variability in terms of participants’ initial fine motor skills at pre-test, despite being matched for age, gender and general cognition. As a result, participants for the current study completed the Disabilities of Arm, Shoulder and Hand (DASH) questionnaire (Hudak et al., 1996) prior to pre-testing. Participants were randomly allocated to either an active experimental group (*n* = 9) or a waitlisted inactive control group (*n* = 8), matching as much as possible for DASH score, age and gender.

Two participants withdrew after pre-tests had been conducted: one participant from the waitlisted control group could not attend the training program on the day of the week available and so chose to withdraw, the other participant from the experimental group withdrew after completing the training program but did not complete post-testing. One participant in the waitlisted control group withdrew after completing post-testing but before receiving the training program. This participant’s pre/post data for the control period was retained.

The remaining participants in each group who completed both pre and post testing did not significantly differ in age [*t*(13) = −0.72, *p* = 0.48, CI difference = −7.48, 3.73], or DASH score [*t*(13) = 0.62, *p* = 0.55, CI difference = −4.08, 7.35].

Participants in each group (experimental or waitlisted control) were then allocated to four separate classes (two for the experimental groups, E1 and E2, two for the control groups, C1 and C2, each group with a maximum of *n* = 4) depending on convenience in terms of the timing of the lessons each week. The distribution of males to females^3^ for each of these classes was 1:3 with the exception of class C2 where the male participant assigned to this class withdrew from the study.

Ethics approval was obtained from the Human Ethics Committee at Western Sydney University. Information sheets were distributed to participants on initial contact with the research team. Capacity to consent was assessed during initial contact with participants when arranging baseline testing sessions (email and phone), and at baseline testing when participants were asked to confirm their understanding of the study by explaining the key points in their own words. Participants were informed they had the right to withdraw at any time without consequence. Continuing consent was assessed each week in the training program, with the opportunity to discuss any questions surrounding the research project in each training session and in pre and post testing. Anonymity was ensured by collecting data in a re-identified manner (using participant codes): this was chosen to allow the different data types for one participant (paper-based, computerized files) to be linked. Interview data was not anonymous on collection on the completion of the experiment (collected by the first author), but transcripts were stored in a de-identified manner for analysis (by first and second authors). The second author’s dual role as researcher and facilitator posed certain ethical issues surrounding the anonymity of participants and the subjectivity of analysis that were addressed as such: (1) The facilitator was not involved in any of the recruitment or consenting procedures with the participants, nor any of the data collection sessions (pre-/post-testing and interviews), (2) The facilitator only had access to the de-identified interview statements as part of the analysis, (3) The facilitator and first author independently analyzed the qualitative data and agreed on revised themes.

### Training Program

The same single facilitator delivered all of the lessons in the 10-week training program to all of the participant groups. The training program consisted of 10 lessons, each of 60 min duration, supplemented by at-home practice specified at 30 min per day (total intervention duration was approximately 600 + 1800 min). The time taken for at-home practice was monitored through participant practice diaries (see section Practice Diaries). All lessons incorporated three main elements to a varying degree: (i) exercises/warm-ups, (ii) playing of melodies, and (iii) ensemble playing tasks. Earlier lessons had an emphasis on exercises and melodies, with the later parts of the program more strongly emphasizing ensemble tasks. Exercises and warm-ups involved simple sequences to familiarize participants with the notated symbols, different pitches and durations of notes. Melodies were planned to be increasingly complex along the 10-lesson program, with increasing duration (number of notes), pitch range and rhythmic complexity. Difficulty was also scaled in terms of hand position required to play the melody, e.g., melodies in the beginning of the program had a narrow pitch range and could be achieved by the hand staying in one position on the piano. Melodies with greater difficulty often involved having to change hand position. Ensemble tasks involved learning different parts (melody plus one of chords, bass line or a counter-melody), and playing these either in unison, or as part of a turn-taking exercise. More than one type of ensemble task could be included as part of any one song.

All materials in the program were delivered using the simplified notation system *Figurenotes*. *Figurenotes* was developed in Finland in the mid-1990s, designed primarily to increase accessibility to learning and performing music through lowering the cognitive demand associated with learning and processing musical notation (Kivijärvi, 2019)^4^. The use of *Figurenotes* in educational and therapeutic settings is now spreading beyond Finland (Criswell, 2014), and is being found effective with people from a diversity of age groups and functional abilities (Ruokonen et al., 2012).

*Figurenotes* is a concrete, matching system where the pitch and octave of a musical note are represented by a figure of a specific color and shape respectively. The colors and shapes of the visual symbols on the score are exactly matched by stickers applied to the keys on the instrument being used. Note duration is depicted by the horizontal length of the figure, with a note two beats long in duration being depicted twice as wide as a one-beat note, and so on. The tasks required of playing from traditional notation involve (i) reading a symbol, (ii) converting that symbol, and (iii) using the appropriate motor action to play the required note. Using a simplified notation system such as *Figurenotes* aims to reduce the conversion step needed so that it becomes a matching task.

Repertoire was initially selected by the facilitator to include simple exercises, children’s (nursery rhymes) and adult’s material (e.g., a mixture of Western popular songs and well-known classical melodies). Where possible in the training program, well-known songs were presented with lyrics in order to prompt participants’ memory of the melody, and to aid in recall when playing. Participants were asked for their selections of repertoire early on in the program, and these choices were incorporated for the classes in later weeks when possible.

### Screening Measures

#### Addenbrooke’s Cognitive Examination III Mini Version (M-ACE)

The mini version of the Addenbrooke’s Cognitive Examination-III assesses cognitive performance in terms of attention, memory, verbal fluency, language and visuospatial abilities (Hsieh et al., 2015) and provides high diagnostic accuracy for screening of Alzheimer’s disease (Matiás-Guiu et al., 2017) and other dementias (Hsieh et al., 2015). Scores are summed across various tasks to give a total out of 30, with higher scores reflecting better cognitive performance. Two cut-offs are identified in Hsieh et al. (2015) for the use of screening participants for research, the upper being a score of 25, and the lower being a score of 21. A specificity of 1 is obtained at this lower cut-off. All participants in the current study achieved a score of 25 or above.

#### Disabilities of Hand, Shoulder, and Arm Questionnaire (DASH)

The DASH (Hudak et al., 1996) is a 30-item self-report questionnaire that asks participants to rate the severity of symptoms in their shoulder, arm and hand (e.g., weakness, tingling or pain) and abilities to perform a variety of activities of daily living (e.g., preparing a meal, carrying a shopping bag or briefcase, or pushing open a heavy door) in the past week. The DASH has high internal consistency (Cronbach’s alpha = 0.96) (Kennedy et al., 2011). High reliability and validity has been demonstrated in patients with disorders of upper extremities (Turchin et al., 1998) as well as cross-culturally (Atroshi et al., 2000).

### Pre-/Post-cognitive and Motor Tests

All cognitive and motor tests were assessed both at pre and post-test, and were administered by a member of the research team blinded to group allocation^5^ ^,6^. It should be noted that the final set of post-tests administered to the waitlist control group upon completion of the training program would have identified these participants to the researcher as being part of the control group, so the process cannot be considered fully blinded.

#### Trail Making Test (TMT)

The Trail Making Test assesses participants’ visuo-motor skills, sequencing, processing speed and cognitive flexibility (Bowie and Harvey, 2006). Part A consists of 25 numbered circles on a piece of paper which participants have to join up in consecutive order by drawing a line with a pencil. Part B is more challenging in terms of executive control and visual search: it consists of 25 circles including both numbers and letters. Participants have to draw lines between them in ascending order alternating between the numbers and letters. The TMT Part A has been validated primarily for visuoperceptual abilities and Part B for working memory and cognitive switching (Sánchez-Cubillo et al., 2009), although is susceptible to practice effects in short time periods (Bowie and Harvey, 2006). Scores are the time taken to complete parts A and B. Additionally, the difference score *delta* (time for part B – time for part A) attempts to isolate the part of the score attributable to performance on cognitive switching.

#### Jebsen Taylor Hand Function Test (JTHFT)

The Jebsen Taylor Hand Function Test is an assessment of a range of uni-manual tasks that are required in activities of daily living (Jebsen et al., 1969). This test shows high interrater reliability (*r* = 0.82–0.91) and high stability, particularly for adults aged > 60 (*r* = 0.84–0.85) (Poole, 2011). The Jebsen Taylor Hand Function test comprises a collection of seven activities carried out by the participant’s non-dominant hand, and subsequently by their dominant hand: (i) writing, (ii) simulated page turning, (iii) lifting small objects, (iv) simulated feeding, (v) stacking, (vi) lifting large, lightweight objects, and (vii) lifting large heavyweight objects. Each task is timed, and the times for all activities are summed into a total score, one each for the participant’s non-dominant and dominant hand.

#### Visuomotor Synchronization Task

Participants completed a visuomotor synchronization task to assess fine motor function and visuomotor coordination (Varlet et al., 2014). This task involved the presentation of a visual pacer (a red circle) on a computer screen, which would periodically oscillate across the horizontal axis at a frequency of 1 Hz for a duration of 60 s. Participants were asked to synchronize the movements of their right forearm (index finger extended) with the onscreen pacer. Their 3D movements were captured using the Polhemus wired motion capture system with a motion tracking sensor attached to the tip of their right index finger. Further details on the method of this task and calculation of outcome measures can be found in Varlet et al. (2014). The test used in the current study had two conditions pertaining to how the position of the finger sensor was mapped onto an onscreen visual circle reflecting the participant’s movement: (1) real-time, and (2) a delayed condition where mapping was delayed by either 3 or 4 samples, equating to a relative phase offset of approximately −36 degrees or −48 degrees respectively. These two delayed levels were counterbalanced across participants, and across pre- and post- testing such that one participant never received the same delayed condition for both. After two practice trials, participants completed 12 experimental trials (6 each for the real-time and delayed conditions). Results were calculated as the mean relative phase in degrees for each condition (normal or delayed), where 0 degrees was a perfect synchronization onscreen between the participant’s visual circle and the oscillating pacer, and 180 degrees was an anti-phase movement of the participant’s visual circle in comparison to the oscillating pacer.

### Facilitator Observations

The facilitator responsible for delivering the music instrument training program recorded observations of each class after each weekly lesson. The facilitator decided on the types of issues to record from week to week depending on the events in each particular lesson. Observations included particular issues or noteworthy events (positive or negative) that were either individual or group concerns, e.g., aptitude, engagement level, perceived difficulty of tasks or progression in acquiring musical skill.

### Practice Diaries

Participants were asked to keep daily practice diaries according with their at-home practice schedules. Paper forms were given to the participants at the beginning of the training program with space to record details on the exercise/song being practiced, and an approximation of the time spent on each. These were collected each week by the facilitator, with some participants opting to submit all practice diaries on the conclusion of the program. Although no space was given for participant comments, a few qualitative statements were recorded on the practice diaries. These were included in the qualitative analysis^7^.

### Post-program Semi-Structured Interview

Participants were invited to complete a semi-structured interview over the phone after completing the training program. Interviews were conducted at a different time and in a different format to the post-testing sessions in order to preserve blinding of the assessor. Six of the participants completed this stage.

Seven questions were based on Brookfield’s Critical Incident Questionnaire (Brookfield, 1995), for example, asking participants to reflect on moments when they were most engaged, or most distanced from the activities. A further nine questions asked participants their opinions on a variety of aspects of program design including (i) the choice of repertoire, (ii) being able to practice with a piano at home, (iii) the use of *Figurenotes* as a notation system, and (iv) whether participants had noticed any differences in their musical or other skills as a result of the program.

### Data Analysis

The current study involved quantitative data analysis from the general cognitive and motor skills tests, as well as qualitative data analysis from the collected observations, practice diaries and interviews. Quantitative analysis was first conducted on the collected data to test the main hypothesis that training would lead to improvements in cognitive and motor skills (confirmatory analyses), with further analyses informed by qualitative data results concerning potential class differences (exploratory analyses).

#### Quantitative Data Analysis

This paper reports quantitative analyses for all tests which included a physical fine motor component [Trail Making Test (TMT), Jebsen Taylor Hand Function Test (JTHFT), and Visuomotor Synchronisation Task]. This comprises a total of seven different measures, denoted *JTHFT_dom, JTHFT_non_dom* (from the Jebsen Taylor Hand Function Test), *TMTA*, *TMTB*, *TMT_delta* (from the Trail Making Test), and *Polhemus*, *Polhemus_del* (from the visuomotor synchronization task). For each type of measure, a Bayesian regression model predicted the post-test score (dependent variable) using a number of predictors (independent variables). To facilitate understanding of these variables, Figure 1 shows how the tests undertaken in the study were designated as pre-tests and/or post-tests. Note that the experimental group had a total of two sets of tests, while the waitlisted control group had a total of three sets of tests. For the latter group, the second set of tests served as both a post-test (with respect to the first set of tests) and as a pre-test (with respect to the third set of tests).

The regressions were all Bayesian. An advantage of Bayesian regression is that, given the observed data and a prior distribution (see the next paragraph for a discussion of priors), it calculates the whole *posterior probability distribution* of each effect rather than only a point-estimate of the most probable effect of each predictor. This allows for *credibility intervals* to be calculated; unlike the confidence intervals in classical regression, credibility intervals have a straightforward and intuitive meaning: given the model and the data, the 95% credibility interval of an effect is the interval we can be 95% certain contains the effect’s true value. It also allows *evidence ratios* to be calculated; these are probability ratios (odds) in favor of directional hypotheses (such as a given effect being greater than zero). Due to our use of a Bayesian approach, we do not report classical *p*-values; instead, we focus on effect sizes and evidence ratios.

Another advantage of Bayesian regression is that we can use *weakly informative priors* (Gelman et al., 2014), which formalize sensible expectations of the range of effect sizes and, in so doing, minimize the possibility of erroneously large effect sizes such as may result from fitting small or noisy data sets. Given that all the data were standardized before entering into the models, this can be captured with a student t prior with 3 degrees of freedom, a mean of zero, and a scale of 1; this encodes our prior belief that the most likely effect (β) of each predictor is zero (i.e., the “null” hypothesis of no effect) but also allow for effects of a reasonable size – small to medium effects are likely, large effects are less likely, extreme effects (i.e., effects with magnitudes greater than 3) are highly unlikely. This prior was used for the effect of each predictor in each model. The models were run in the R package brms (Bürkner, 2017, 2018), which is a high-level interface for Stan – an open source platform for full Bayesian statistical inference with MCMC sampling (Carpenter et al., 2017). For the models which used random effects, the brms default prior for random effects was used; all priors are detailed in Supplementary Materials.

The predictors used in the regression models were:

• *test*_*pre* ∈ *ℝ* is the pre-test score for the respective outcome measure.
• *is*_*trained* ∈ {0, 1} is a 2-level categorical variable indicating whether the participant has completed the training program at the time of the post-test. This variable can be only 1 for the experimental group; 0 or 1 for the waitlisted control group.
• *n*_*pretests* ∈ {0, 1} is a 2-level categorical variable indicating whether the participant had previously undertaken one pre-test at the time of their post-test (coded 0), or whether they had previously completed two pre-tests at the time of their post-test (coded 1). This variable can be only 0 for the experiment group, 0 or 1 for the waitlisted control group.
• *age* ∈ *ℝ* is the age of the participant.
• *DASH* ∈ *ℝ* is the DASH score obtained as a screening measure (detailed in section Disabilities of Hand, Shoulder and Arm Questionnaire (DASH)).

Using the pre-test score as a predictor of the post-test score is a flexible alternative to simply regressing the difference between the pre- and post-tests (Gelman and Hill, 2007); we would expect this predictor to have a strong positive impact on post-test score. The categorical variable *is_trained* is of key interest here – it allows us to assess whether the treatment has had an impact on the post-test score, relative to no treatment. The number of pre-tests undertaken by each participant, for a given post-test, controls for possible improvements gained by additional practice or, in the opposite direction, for an accelerating performance decline over the time period of the experiment. *Age* and *DASH* are covariates that are not of direct interest; they are included because they are pre-treatment variables that can control for any possible imbalance across the groups.

With the exception of the Polhemus post-test scores, all continuous variables were standardized. Linear models were used for JTHFT and TMT scores. The standardizations mean that these models’ coefficients represent standardized effect sizes; for example, the coefficient for *is_trained* estimates the number of standard deviations by which the post-test changes after training. The Polhemus data are phase angles, hence lie in the interval [−180, 180) degrees; the absolute value of these were taken because we are interested in *phase error*, and the resulting values were divided by *180* to put them into the unit interval [0, 1); hence the units of phase error are, here, *half-turns*. Beta regression with a logit link function is appropriate for data in the unit interval. The logit link function means that interpretation of the coefficients (effects) is more complex than in the linear case: given an *is_trained* coefficient of β, the untrained phase error ϕ_*0*_ is multiplied by $\frac{\exp\left(\beta_{1}\right)}{\phi_{0}\,\exp\left(\beta_{1}\right)-\phi_{0}+1}$ after training. This means that the change in the phase error, after training, depends not just on the coefficient but also on the untrained phase error. In general, coefficients greater than 0 increase the untrained phase error; coefficients below 0 decrease the phase error. Supplementary Figure 2 shows how a unit increase in a predictor changes phase errors as a function of its original value and of its estimated coefficient β.

For each of the seven types of measure, four regression models were compared – using approximate leave-one-out cross-validation (Vehtari et al., 2017) – to assess their ability to predict out-of-sample data. A “maximal” model (Barr et al., 2013) had interactions between *is_trained* and *age* and between *is_trained* and *DASH*, and also had a group-level (random-effects) intercept, grouped by participant, to take account of random variation of participants’ abilities in the post-test. This maximal model was reduced in three ways: by removing the interactions, by removing the group-level intercept, by removing both the interactions and the group-level intercept. For every type of measure, the model without the interactions out-performed models with the interactions. In all but one case (the delayed Polhemus measure), the model without the group-level intercept performed best. The reported results are from these best-performing reduced models.

To qualify the evidence for or against any given hypothesis (e.g., that an effect is greater or less than 0), we followed guidelines (proposed by Jeffreys, 1961 as cited by Dienes and Mclatchie, 2018; Kruschke and Liddell, 2018), which state that evidence ratios of 1–3 represent no evidence for the tested hypothesis; evidence ratios of 3–10 are “moderate” evidence for the hypothesis; evidence ratios of 10–30 are “strong” evidence; and evidence ratios above 30 are “very strong” evidence. The inverse of these ratios reflect evidence against the tested hypothesis; that is, evidence ratios of 1/3–1/10 show moderate evidence against the hypothesis, ratios of 1/10–1/30 show strong evidence against the hypothesis, and so on. Note that these hypotheses test for an effect’s direction – the evidence ratio is the odds in favor of an effect being positive rather than negative (or vice versa). Although a type of Bayes factor, these directional tests are not equivalent to Bayes factors that assess the evidence for or against the point-null hypothesis of precisely zero effect.

#### Qualitative Data Analysis

Qualitative statements were integrated from the two main sources at the point of analysis, (i) facilitator observations, and (ii) participant post-program interview transcriptions, to allow for a multi-viewpoint assessment of the training program. Thematic analysis (Braun and Clarke, 2006) was performed using the following procedure: (1) familiarization with the data, (2) generating initial codes, (3) searching for themes, (4) reviewing themes, (5) defining and naming themes, and (6) producing a report. Steps 1-4 were performed independently by the first and second authors. The revised themes and subcategories were then agreed between the two coders, and Steps 5–6 completed. Codes generated for the qualitative statements retained an indicator of the data source (participant or facilitator).

## Results

### Confirmatory Analyses of Quantitative Results

Confirmatory analyses were run with Bayesian regression models (with group-level effects, where desirable) to test the effects of the music training intervention on the outcomes of participants’ motor skill tests, controlling for the number of pre-tests the participant had conducted, their age and their DASH score. For each outcome measure, estimated effects, standard errors, 95% credibility intervals, and evidence ratios from hypothesis tests on the effect of training are reported in Table 1. Full summaries of each model are included in Supplementary Materials.

**TABLE 1:** Hypothesis tests for the effect of training.

**Test**	**Hypothesis**	**Estimate**	**Est.Error**	**CI.Lower**	**CI.Upper**	**Evid.Ratio**	**Post.Prob**
JTHFT_dom	(is_trained1) < 0	−0.13	0.34	−0.68	0.43	1.86	0.65
JTHFT_non_dom	(is_trained1) < 0	−0.25	0.48	−1.04	0.53	2.34	0.70
TMTA	(is_trained1) < 0	−0.30	0.45	−1.02	0.44	3.03	0.75
TMTB	(is_trained1) < 0	0.48	0.48	−0.31	1.26	0.17	0.15
TMT_delta	(is_trained1) < 0	0.51	0.49	−0.30	1.32	0.17	0.15
Polhemus	(is_trained1) < 0	−0.15	0.44	−0.88	0.56	1.77	0.64
Polhemus_del	(is_trained1) < 0	0.28	0.51	−0.53	1.12	0.41	0.29

There is no evidence that training has either a positive or a negative impact on either of the Jebsen Taylor hand function tests. There is moderate evidence that training has an impact on all three trail making tests; however, although the impact is positive for part A (effect = −0.3 = −1.9 s, evidence ratio = 3.03), it is negative for part B (effect = 0.48 = 11.8 s, evidence ratio = 0.17) and their difference score (effect = 0.51 = 11.8 s, evidence ratio = 0.18). There is no evidence that training has either a positive or a negative impact on either of the Polhemus tests.

### Qualitative Results

Six main themes were created from the collated qualitative statements made by the facilitator (facilitator notes) and the participants (practice diaries and post-program interviews): *enjoyment, intellectual challenge, lessons, practice, groups, and benefits.* These themes, their associated sub-themes and example statements from each are listed in Tables 2–6 along with the data source (participant or facilitator). The majority of themes included statements taken from both the participants and the facilitator (*enjoyment, intellectual challenge, practice, groups)*. Participant-only statements appeared for *lessons* and *benefits* sub-theme (general). Facilitator-only statements described differences between individual participants and the classes (*group* sub-theme *differences)*.

**TABLE 2:** Sub-themes and example statements from theme of *Enjoyment*.

**Theme**	**Sub-themes**	**Sources: Participant (P) and/or Facilitator (F)**	**Example statements**
Enjoyment	General	P	*“I thoroughly enjoyed all the lessons” (P)*
	Aesthetic	F	*“They seemed to like the way the bass line was like another melody, and they understood how it fitted with the melody – showing quite a degree of musical sophistication actually” (F)* *“Added harmony part to Twinkle Twinkle today*…*it sounded GREAT, and they commented on this” (F)*
	Motivation	P, F	*“When you’re playing something like Ode to Joy you can hold your head up and say Oh, I’m playing Beethoven” (P)* *“All excited for Happy Birthday – this seems to be the one they can use to connect with others, show off” (F)*

**TABLE 3:** Sub-themes and example statements from theme of *Intellectual Challenge*.

**Theme**	**Sub-themes**	**Sources: participant (P) and/or facilitator (F)**	**Example statements**
Intellectual Challenge	*Figurenotes*	P	*“not having to learn notes I found it much easier than I was expecting it to be” (P)* *“[Figurenotes] facilitated an easy and fast entry into being able to play music immediately” (P)*
	Repertoire	P, F	*“because the music was familiar it gave people the confidence to try it” (P)* *“I think adult repertoire should be more different to children’s music”(P)* *“there was such a great variety of tunes, there was none we disliked. Some were a little bit harder to play than others but there were no problems” (P)* *“all groups (experimental) achieved it well and seemed pleased. Good to push them a little out of their comfort zone!” (F)*

**TABLE 4:** Sub-themes and example statements from themes of *Lessons* and *Practice*.

**Theme**	**Sub-themes**	**Sources: participant (P) and/or facilitator (F)**	**Example statements**
Lessons	Facilitator	P	*“I enjoyed the knowledge, teaching attitude and support of [the facilitator]” (P)* *“[the facilitator] has a great attitude to learning and understanding people who may have difficulties with learning, was very gentle and patient” (P)*
	*Figurenotes* sustainability	P	*“obviously its hard to get more of that style of lesson” (P)* *“I would have liked a follow on from Figurenotes transferring to normal notation” (P)*
Practice	Motivation	P	*“Consistent practice produced improved playing and memory” (P)* *“[I enjoyed practicing]. I felt I was achieving something”(P)*
	Quality	F	*“Initially, they did not have much idea how to practice” (F)* *“Learning HOW to practice is interesting for them” (F)*

**TABLE 5:** Sub-themes and example statements from theme of *Groups*.

**Theme**	**Sub-themes**	**Sources: participant (P) and/or facilitator (F)**	**Example statements**
Groups	Social	P	*“From lesson 1 the teacher and the group bonded, we were all keen to help each other” (P)* *“[I was surprised by] the general enthusiasm of the group itself and the way we came together to play as a group”(P)* *“In a group situation you’re a little bit nervous that you don’t make a fool of yourself” (P)* *“[I disliked] the other participants not listening to instructions!” (P)*
	Playing	P/F	*“There’d be two of us playing the chords and two of us playing the melodies – it was great.” (P)* *“One thing I enjoyed was playing with the group – the interaction with the other players”(P)* *“Participants enjoyed playing all the ensembles in this lesson (as they have in previous lessons)*…*In contrast, playing the same melody altogether sometimes caused friction”(F)*
	Differences	F	*“Some clear differences between the groups are emerging.* < *Class* > *are very switched on*…*participating with understanding and almost teaching themselves. They get on very well together and this may help.” (F)* *“* < *Class* > *are learning well but they always seem to need me to prompt them and explain things for them*…*no one is taking initiative in the group.”(F)* “< *Class* > *are not cohesive, connected” (F)*

**TABLE 6:** Sub-themes and example statements from theme of *Benefits*.

**Theme**	**Sub-themes**	**Sources: participant (P) and/or facilitator (F)**	**Example statements**
Benefits	Dexterity	P, F	*“I think my dexterity improved in my fingers” (P)* *“I felt it was very beneficial physically and mentally” (P)* *“No, I’ve got pretty good manual dexterity anyway. I couldn’t notice any marked difference”(P)*
	Self-efficacy	P	*“The skill I’d learned – the sound that I was producing was coming from me” (P)* *“I did feel increased self-esteem and belief that I can try new things and expect to achieve well” (P)*
	Others	P	*“My memory and concentration have improved” (P)*

#### Enjoyment

Table 2 describes the main theme of *enjoyment* with sub-themes and sample statements provided. Participants clearly enjoyed the training program in general, and the facilitator noted the participants’ sophisticated appreciation of aesthetic aspects of the sounds produced (e.g., when combining ensemble parts like a melody and bass line in comparison to unison playing). Participants’ enjoyment was directly connected to their motivations for learning to play such as being able to learn a skill and/or repertoire that held a certain level of esteem (e.g., Beethoven’s Ode to Joy), or was familiar enough to everyone to be able to be “shown off” at certain occasions (e.g., Happy Birthday).

#### Intellectual Challenge

Table 3 describes the main theme of *intellectual challenge* with sub-themes and sample statements provided. The choice of notation system and selection of repertoire influenced how intellectually challenging the tasks were. Participants saw *Figurenotes* as a simple and approachable form of notation that allowed quick and easy entry into playing melodies from the first lesson. Familiarity with the chosen repertoire may have also aided the early success, as some participants noted that most of these songs were “in their head” already and that the notation simply provided a support. Although participants found tasks involving playing chords and melodies simultaneously quite complex, participants appeared to enjoy the results of being able to play these more challenging pieces. The variety of repertoire ensured that most participants enjoyed at least some of the songs (if not all), and also provided a range of difficulty levels.

#### Lessons and Practice

Table 4 describes the two main themes of *lessons* and *practice* with sub-themes and sample statements provided. Participants noted the importance and efficacy of the facilitator in this program, commenting on his supportive attitude and clear direction. Several participants expressed interest in continuing training after the program’s conclusion, but were concerned about the sustainability of using the *Figurenotes* notation. They asked for further *Figurenotes* resources, and also how to transition from *Figurenotes* to traditional notation. In terms of practicing between the lessons, participants all noted the importance of practicing and found this gave them continued motivation and enjoyment to consolidate the learning that had taken place that week. However, the facilitator noted that further instruction was often required in how to practice.

#### Groups

Table 5 describes the main theme of *groups* with sub-themes and sample statements provided. In general, the group format of lessons was a source of stimulation for participants’ progression, but was also at times a cause for frustration. From a social aspect, participants noted the general group enthusiasm and a keenness to assist one another. However, negative contributions to the social dynamic of the group included frustrations with others seemingly not paying attention, and anxiety as some participants noted that they were afraid to play incorrectly. Comments from both the participants and facilitator suggested that participants found the group format beneficial, especially when playing together as an ensemble. Participants enjoyed this group playing, particularly when this involved playing different parts at the same time – what could be termed co-operative ensemble playing. Both the facilitator and participants noted that participants expressed irritation with one another when playing together in unison, particularly when their co-performer would be playing at the wrong tempo. Differences between the classes were noted by the facilitator: some well-motivated participants in one of the classes appeared to initiate their own learning (self-directed), whereas other classes looked for more facilitator-led instruction. Facilitator comments were also made on the presence or lack of group cohesion.

#### Benefits

Table 6 describes the main themes of *benefits* with sub-themes and sample statements provided. Both the facilitator and the participants noted an improvement in manual dexterity. However, this was not universal as some participants noted that they had a good level of dexterity on entry to the program, and so did not notice much of a difference. Other benefits reported by the participants included increased self-awareness and self-efficacy. Many participants expressed that they had initially been unsure of being able to learn a musical instrument and were surprised by how far they could progress in such a short period of time. Other participants noted that they had seen changes in their memory and concentration but again this was not reported universally across all participants.

### Exploratory Analyses of Quantitative Results

In keeping with the mixed methods explanatory sequential design of this study, exploratory analyses on the data were conducted for potential influences identified as part of the qualitative analysis. The facilitator’s perception of particular class differences in cohesion and amount of self-directed learning was used as motivation to directly examine the effect of class (C1, C2, E1, or E2) on the motor skill outcomes. The models can only test directly for differences between the impacts of training on groups C1 and C2 (comparing scores from the second and third sets of tests, see Figure 1).

This was achieved by running the models described in section Confirmatory Analyses of Quantitative Results but with an additional 4-level factor denoted *class*, indicating the class (C1, C2, E1, or E2) each participant belonged to (C1 was the reference level and treatment coding was used). This *class* predictor interacted with *is_trained*, enabling an assessment of whether or not the impact of training differed between classes. Due to the inclusion of this interaction, the *n_pretests* variable is removed to avoid a rank-deficient design matrix. For each outcome measure, Table 7 reports the estimated effects, standard errors, 95% credibility intervals, and the evidence ratios from hypothesis tests on the difference of the training effect between groups C1 and C2.

**TABLE 7:** Hypothesis tests on differences of the effect of training between classes C1 and C2.

**Test**	**Hypothesis**	**Estimate**	**Est.Error**	**CI.Lower**	**CI.Upper**	**Evid.Ratio**	**Post.Prob**
JTHFT_dom	(is_trained1:classC2) < 0	−0.29	0.56	−1.21	0.62	2.42	0.71
JTHFT_non_dom	(is_trained1:classC2) < 0	0.45	0.68	−0.63	1.59	0.33	0.25
TMTA	(is_trained1:classC2) < 0	−1.21	0.80	−2.54	0.06	15.91	0.94
TMTB	(is_trained1:classC2) < 0	0.12	0.76	−1.10	1.37	0.78	0.44
TMT_delta	(is_trained1:classC2) < 0	0.31	0.78	−0.94	1.61	0.52	0.34
Polhemus	(is_trained1:classC2) < 0	0.32	0.68	−0.77	1.45	0.46	0.32
Polhemus_del	(is_trained1:classC2) < 0	0.58	0.66	−0.48	1.69	0.23	0.19

The hypothesis tests show strong evidence that – for trail making part A – class C2 benefitted more from training than did class C1 (effect = −1.21 = −7.8 s, evidence ratio = 15.91). However, there is moderate evidence that – for the Jebsen Taylor non-dominant hand measure and the delayed Polhemus measure – class C2 benefitted less from training than did class C1: respectively, *JTHFT_non_dom* effect = 0.45 = 5.3 s, evidence ratio = 0.33; *Polhemus_del* effect =0.58, evidence ratio =0.23. See Supplementary Figure 2 for a plot showing how to interpret effects in the Polhemus models. Supplementary Figures 3–5 show plots of the training by class interactions for these three measures (Jebsen Taylor non-dominant hand, Trail Making Test Part A, and the Polhemus delayed condition). For the remaining four measures, there is no evidence of any difference between the impact of training on the two classes.

## Discussion

Our main hypothesis was that results of motor skills tests would improve as a result of a 10-week piano training program. We found moderate evidence to support hypothesized gains in part A of the Trail Making Test suggesting that training positively affected the participants’ visuo-motor skills. This confirms the similar effect seen by Seinfeld et al. in a 4-month piano intervention with weekly 90 min group lessons (2013). We found moderate evidence for negative impacts of training on part B of the Trail Making Test (and difference score delta), suggesting that our participants did not benefit from piano training in terms of more challenging executive function such as cognitive switching. This is in contrast with Bugos and colleagues’ (2007) positive results from a 6-month piano intervention with weekly 30 min individual lessons. Our training program at 10 weeks length may simply not be enough to see benefits above simple visuo-motor skills. However, our results show a performance decrease in TMT-B rather than performance maintenance. As the TMT-B is scored solely by completion time, we cannot disentangle potential reasons behind the decreased performance for our participants, which could include a change in participant’s motor skill, or a modified trade-off between performance speed and accuracy (Shmuelof et al., 2012). Beginner-level piano playing tasks emphasize pitch accuracy over speed (i.e., playing the correct sequence over playing the sequence at a set speed): it may be that within this short training period, participants have developed skills to play simple sequences (which transfers well into the numbers-only sequence of the TMT-A), but tend to approach more complex sequences (like the alternating number-letter sequence of the TMT-B) more carefully, prioritizing accuracy. Further research examining the development of transferred motor skills for complex sequences over several timepoints would be needed to support this explanation. We did not see hypothesized gains in the performance of daily fine motor tasks as assessed by the Jebsen Taylor Hand function tests and the visuo-motor coordination task. The transfer of skills into general fine motor function may require longer training times and, in the absence of follow-up testing, it is difficult to estimate how long these particular skills would require for consolidation. Following on from Schneider and colleagues’ recommendation to determine the level of musical exposure required to protect older adults from cognitive decline (Schneider et al., 2018), it appears that a short-term intervention (10 weeks) constitutes enough training to see positive benefits in simple visuo-motor skill. However, the question remains for older adult novices: what are the specific effects of the length of training, and when is the best time for an older adult to commence to gain optimal protective cognitive and motor benefits?

Another explanation for our results could be the potentially reduced cognitive load associated with using the *Figurenotes* notation system for the 10-week training program in place of traditional music notation. Difficulties in learning to read traditional notation are often cited by children and young adults as a reason for ceasing musical training (McPherson, 2005), and notation appears to be redundant at least for solving rhythmical problems (Owens and Sweller, 2008). However, it may be that the higher cognitive effort involved in learning to read traditional notation creates a more cognitively demanding task, and could lead to added cognitive benefit. Further research is required that establishes (i) the cognitive demand associated with different forms of musical notation (and the absence of notation), and (ii) the cognitive gain that is produced as a result of learning a musical instrument with each notation type.

From our qualitative analyses, we demonstrated that older adults have a range of motivations for taking up a musical instrument, and for these novices, the ability to connect with others, and learn a respected skill is important. Learners require a balance between motivation, the challenge, and the resources available to meet those challenges, much like we see in other musical groups for older adult novices (Davidson et al., 2014; Roulston et al., 2015; Lamont et al., 2018), and in older adults who have continued music playing long-term (MacRitchie and Garrido, 2019). Our qualitative results highlighted the high level of musical sophistication that older adult novice learners could bring to music lessons, and their aspirations to create beautiful sounding music. Aesthetically speaking, ensemble playing provides good opportunity for novice learners to create complex sounds together, and this was an aspect of the lessons particularly enjoyed by the participants. Students also often want to be able to play particular repertoire that holds esteem for their friends and relatives, or opportunities to share their new skills on family occasions (birthdays), or being able to connect with younger generations (e.g., playing with grandchildren).

Participants particularly noted their feelings of achievement, competence and increased self-efficacy after the training program; this aligns with self-determination theory (Krause et al., 2019), providing further evidence that music training programs may be linked to increasing wellbeing for older adults. Previous research has noted music-specific self-efficacy increases after short, intense training programs (2 weeks), noting that longer-term programs may be required for transfers to general self-efficacy (Bugos et al., 2016). After a 10-week program, our participants stated that they felt able to play the musical instrument (music-specific self-efficacy), but also made statements regarding their general ability to learn new skills, although this was not measured quantitatively. Our qualitative statements suggested that perceived progress was noted through individual practice, as well as the review of learned songs each week in the lesson structure. Further quantification of older adults’ self-efficacy both in domain-specific (music) and general terms after such a short-term music training intervention would be required to determine whether general gains could be made in this short time-frame.

Our exploratory analysis confirmed that there were differences in the impact of training on post-test measures (TMTA) between classes (C1 and C2). One main difference in the makeup of these two classes is the lack of a male participant in class C2. The study also did not collect information on participants’ education level or past/current professions which may have an impact on their underlying cognitive and fine motor abilities. However, by including each participant’s pre-test score, each model reported in the results fully accounts for any individual differences in underlying cognitive abilities relevant to the test being modeled. An alternative explanation is that group dynamics may play a role in the extent of motor skills development of the individual group members, although the current study did not collect any data to support this hypothesis. The characteristics behind different group dynamics and their specific effect on cognitive transfer of skills is a point for further research, although previous research would suggest that optimal, cohesive ensemble groups (at least for ensemble rehearsal and performance) are constructed when all participants feel musically ‘adequate’ for the group level and group members are perceived as welcoming and friendly (Pitts et al., 2015; Pitts and Robinson, 2016).

### Limitations

The models in our analyses provided moderate to strong evidence (as reported by the evidence ratios) that the 10-week piano training program affected some of the domain-general fine motor skills outcomes. Although this represents one study in a limited number that quantify the causal effects of music instrument training for older adults, these results must be interpreted with caution. The current study used a waitlisted control design which ensured that all participants received the piano training, however, it was still subject to a small sample size (*n* = 15). This is comparable to Bugos et al. (experimental group *n* = 16, inactive control group *n* = 15) and Seinfeld et al. (experimental group *n* = 13, group experiencing other leisure activities *n* = 16), however, conducting analyses on small amounts of data means it is difficult to find convincing evidence of an effect in either direction. The main results in the current study show moderate evidence of an effect of training (TMTA), and a negative effect of training in part B of the same test. Although these parts of the test reflect differences in the level of executive functioning required to complete the task, taken together, this is still a mixed set of outcomes. As such, we have to be cautious of the evidence shown in cognitive training intervention studies of this size, this being typical of recent research in the music instrument education field. Difficulties with recruitment and retention of older adult novices, and the logistics required to facilitate a facilitator-led music instrument training program for any length of time may contribute to small sample sizes. In spite of this, further research is needed where substantially larger samples of older adult novices can be tracked through a longer-term training program so that we may adequately assess the broad cognitive effects of this type of program.

Out of the two other studies that report general cognitive improvements as a result of piano training programs for older adults, the current study may also be the first to report these results through a quasi-blinded process of assessment (Bugos et al., 2007 – blinding not reported; Seinfeld et al., 2013 – assessor not blinded). However, the final post-test results of our waitlisted control group may still have been subject to assessor bias. It is clear that further testing should be pursued with larger numbers of older adults, and where possible, fully blinded assessments conducted at all time-points. The facilitator observations, particularly regarding the attributes of the individual participants and perceptions of the class dynamics, may also be subject to bias: future research could include an independent observer present throughout the training program to further validate this data.

A strength of the current study is its comparison to participants who are in an inactive control group so we can accurately assess the impact of piano training. However, it is difficult to truly establish a group of older adult participants who are “inactive,” particularly as these were participants who were recruited precisely because they were keen to pick up a new skill. For this reason, using participants as their own controls is perhaps a more reliable comparison, although this has its own limitations in determining whether increases in performance over repeated tests are solely attributable to the intervention. Our study, and specifically our choice of analysis technique allowed this within-groups analysis of the control group participants, while accounting for the between-group differences of the experimental participants. Our analysis also accounted for the number of tests each participant had experienced at the time of the post-test assessment.

In further investigating the possible sources for class differences that lead to differences in cognitive impact of the piano training, our qualitative results demonstrated that differences between classes were apparent concerning the degree of group cohesiveness, as well as the levels of motivation and self-directed learning that took place. This particular result is currently only possible to examine through the lens of the facilitator observations. Further clarification of class differences could be explored via specifically designed measurements of motivation and preferences for self-directed learning before the commencement of the training program, as well as particular questions surrounding participants’ perceptions of cohesiveness of the class at the end of the program.

## Conclusion

The current study provides evidence of mixed results concerning the effects of a short-term (10-week) piano training program on the domain-general cognitive abilities of a group of older adult novices. Positive effects of training were found for Part A of the Trail Making Test assessing participants’ visuo-motor skills. However, negative effects of training were found for Part B and the delta measure of the same test that indicated participants’ cognitive switching skills. Qualitatively, motivation was optimal when all participants were happy with the chosen repertoire (participants were motivated by learning to play music familiar to them) and the groups had formed cohesive bonds. The facilitator observed that groups tended to differ in terms of their cohesiveness, which may have been related to the makeup of individuals’ motivations and degree of self-directed learning. The degree of participant improvement in some of the quantitative measures was predicted by the particular class each belonged to. One explanation of this is that the class itself may impact the cognitive gains that individual participants in that class experience. Further research is required to find the active elements underlying these class differences. These results will make a direct contribution to the evidence base surrounding the use of music instrument training for older adults’ wellbeing and, in particular, give further guidance toward the structuring of group formats for these types of interventions.

## Data Availability Statement

Restrictions apply to this dataset. The datasets for this article are not publicly available because participant information was collected under extended consent procedures for this particular project and similar related projects. Requests to access the dataset should be directed to JM, j.macritchie@westernsydney.edu.au.

## Ethics Statement

The study detailed here involving human participants was reviewed and approved by Western Sydney University Human Ethics Committee. The participants provided their written informed consent to participate in this study.

## Author Contributions

JM and SM contributed to the conception and design of the study. MB organized and delivered the piano training program and conducted facilitator observations. JM and MB conducted the qualitative analysis. JM and AM conducted all quantitative analysis and wrote the manuscript. All authors contributed to manuscript revision, read and approved the submitted version.

## Conflict of Interest

The authors declare that the research was conducted in the absence of any commercial or financial relationships that could be construed as a potential conflict of interest.

## Footnotes

1 As this is the type of music training that has been most tested in both correlational and intervention studies.

2 Formulating the hypothesis in this way also allows for the possibility of practice effects in all tests that may be observed in the control group.

3 Males are generally noted to have an advantage in cognitive performance particularly for visuospatial ability, however, males also appear to experience a steeper rate of decline for these skills (McCarrey et al., 2016). For this reason, the male participants were distributed such that each class initially contained one male.

4 The claim that *Figurenotes* reduces the cognitive load associated with learning an instrument in comparison to traditional music notation has not yet been experimentally tested.

5 The researcher responsible for administering the screening measures and all pre/post cognitive and motor tests was a doctoral student in experimental psychology, and received training on each of the measures from the lead researcher on the project.

6 This paper focuses on the results of all tests with a fine motor skills component. Further tests not reported here were administered assessing working memory (Digit Span test, Wechsler, 1997), mood (Profile of Mood States, McNair et al., 1981), and quality of life (WHO Quality of Life scale, Skevington et al., 2004). The results of these are to be reported in a future manuscript.

7 A very small number of statements were written on the practice diaries (*n* = 2, one statement each from two participants). For this reason, these statements were coded and added to the codes generated from the analysis of the participant post-program interviews.
