# Supplementary material for: Cognitive, Motor and Social Factors of Music Instrument Training Programs for Older Adults’ Improved Wellbeing
Source: Front Psychol. 2020 Jan 10;10:2868. doi: 10.3389/fpsyg.2019.02868 (PMC6968490; doi:10.3389/fpsyg.2019.02868)
Supplement: Supplementary file 1 [file Data_Sheet_1.pdf]

1 Supplementary Materials

1.1 Group distribution

	**Control (n = 7)**	C1 (n = 4)	C2 (n = 3)	**Experimental (n = 8)**	E1 (n = 4)	E2 (n = 4)
No. of males:females	**1:6**	1:3	0:3	**2:6**	1:3	1:3
Age (years)	**69.0 (4.0)**	67.8 (3.7)	70.7 (3.8)	**71.4 (5.3)**	73.0 (5.2)	69.8 (4.8)
M-ACE	**27.4 (1.7)**	27.5 (1.7)	27.3 (1.7)	**29.3 (0.7)**	29.8 (0.4)	28.8 (0.4)
DASH	**31.4 (4.4)**	30.0 (3.7)	33.3 (4.5)	**29.6 (5.0)**	30.6 (3.9)	28.5 (5.7)

1.2 Interpreting effects in beta regression

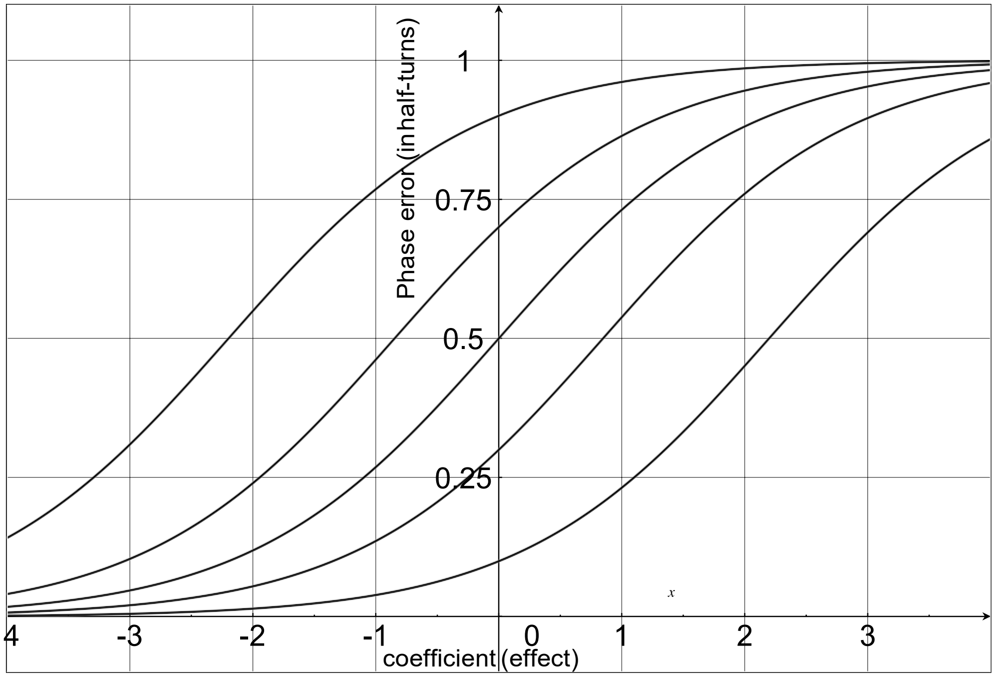

Figure 2. The vertical axis shows the phase error (expressed in half-turn units so it is in the unit interval) after a unit increase in a predictor whose coefficient (effect) is shown in the horizontal axis. The amount of change varies as a function of the coefficient and of the original phase error. The five lines, from bottom-to-top, have original phase errors of 0.1, 0.3, 0.5, 0.7, and 0.9, respectively. Note

that a coefficient of 0 means the predictor has no effect on the phase error; a coefficient larger than 0 increases phase error; a coefficient less than 0 decreases phase error.

1.3 Model priors

1.3.1 Linear models

```
               prior     class               coef
1     student_t(3,0,1)        b
2                             b                age
3                             b            classC2
4                             b            classE1
5                             b            classE2
6                             b               DASH
7                             b        is_trained1
8                             b is_trained1:classC2
9                             b is_trained1:classE1
10                            b is_trained1:classE2
11                            b       JTHFT_dom_pre
12                            b        n_pretests1
13 student_t(3, 0, 10) Intercept
14 student_t(3, 0, 10)     sigma
```

1.3.2 Beta models (for Polhemus)

```
               prior     class               coef
1     student_t(3,0,1)        b
2                             b                age
3                             b            classC2
4                             b            classE1
5                             b            classE2
6                             b               DASH
7                             b        is_trained1
8                             b is_trained1:classC2
9                             b is_trained1:classE1
10                            b is_trained1:classE2
11                            b        n_pretests1
12                            b    Polhemus_del_pre
13 student_t(3, 0, 10) Intercept
14   gamma(0.01, 0.01)       phi
```

1.4 Model summaries

1.4.1 JTHFT_dom model without class interaction

```
Family: gaussian
  Links: mu = identity; sigma = identity
Formula: JTHFT_dom_post ~ JTHFT_dom_pre + n_pretests + is_trained + age + DASH
   Data: fingers_tap_data (Number of observations: 21)
Samples: 4 chains, each with iter = 10000; warmup = 5000; thin = 1;
         total post-warmup samples = 20000

Population-Level Effects:
              Estimate Est.Error l-95% CI u-95% CI Rhat Bulk_ESS Tail_ESS
Intercept        -0.06      0.23    -0.53     0.40 1.00    17600    13567
JTHFT_dom_pre     0.78      0.15     0.48     1.07 1.00    14053    13274
n_pretests1       0.44      0.34    -0.24     1.11 1.00    14209    12546
is_trained1      -0.13      0.34    -0.80     0.54 1.00    12800    13721
age              -0.07      0.16    -0.37     0.25 1.00    13289    11740
DASH             -0.15      0.16    -0.46     0.18 1.00    13045    12966

Family Specific Parameters:
      Estimate Est.Error l-95% CI u-95% CI Rhat Bulk_ESS Tail_ESS
sigma     0.63      0.13     0.44     0.93 1.00    11036    12743


    Estimate   Est.Error      Q2.5     Q97.5
R2  0.6836007  0.08003933  0.4752476  0.7797765

         Hypothesis Estimate Est.Error CI.Lower CI.Upper Evid.Ratio Post.Prob
```

```
1 (is_trained1) < 0    -0.13       0.34    -0.68      0.43        1.86       0.65
2         (age) > 0    -0.07       0.16    -0.32      0.19        0.49       0.33
3        (DASH) < 0    -0.15       0.16    -0.40      0.12        4.71       0.82
4 (n_pretests1) > 0     0.44       0.34    -0.13      1.00        9.00       0.90
```

1.4.2 JTHFT_dom model with class interaction

```
Family: gaussian
  Links: mu = identity; sigma = identity
Formula: JTHFT_dom_post ~ JTHFT_dom_pre + is_trained * class + age + DASH
   Data: fingers_tap_data (Number of observations: 21)
Samples: 4 chains, each with iter = 10000; warmup = 5000; thin = 1;
         total post-warmup samples = 20000

Population-Level Effects:
                    Estimate  Est.Error  l-95% CI  u-95% CI  Rhat  Bulk_ESS  Tail_ESS
Intercept              -0.11       0.32     -0.72      0.54  1.00     16358     15149
JTHFT_dom_pre           0.82       0.17      0.47      1.15  1.00     17713     12409
is_trained1             0.41       0.44     -0.47      1.28  1.00     13115     13081
classC2                 0.14       0.40     -0.65      0.93  1.00     15565     13790
classE1                -0.38       0.86     -2.12      1.31  1.00     13748     10655
classE2                -0.15       0.84     -1.83      1.49  1.00     13209     11673
age                    -0.05       0.17     -0.38      0.28  1.00     16425     13744
DASH                   -0.15       0.17     -0.48      0.20  1.00     15772     13963
is_trained1:classC2    -0.29       0.56     -1.41      0.79  1.00     12833     13368
is_trained1:classE1    -0.36       0.86     -2.11      1.33  1.00     13828     10589
is_trained1:classE2    -0.14       0.85     -1.83      1.54  1.00     13069     11113

Family Specific Parameters:
      Estimate Est.Error l-95% CI u-95% CI Rhat Bulk_ESS Tail_ESS
sigma     0.65      0.14     0.44     1.00 1.00     9862    10207

   Estimate  Est.Error      Q2.5     Q97.5
R2 0.6876289 0.08194121 0.4777197 0.7909059

Hypothesis Tests for class b:
                             Hypothesis Estimate Est.Error CI.Lower CI.Upper Evid.Ratio Post.Prob
1                      (is_trained1) < 0     0.41      0.44    -0.32     1.13       0.20      0.17
2 (is_trained1+is_trained1:classC2) < 0     0.12      0.46    -0.63     0.86       0.64      0.39
3 (is_trained1+is_trained1:classE1) < 0     0.06      0.91    -1.42     1.52       0.90      0.47
4 (is_trained1+is_trained1:classE2) < 0     0.27      0.90    -1.20     1.73       0.59      0.37
5             (is_trained1:classC2) < 0    -0.29      0.56    -1.21     0.62       2.42      0.71
```

1.4.3 JTHFT_non_dom model without class interaction

```
 Family: gaussian
  Links: mu = identity; sigma = identity
Formula: JTHFT_non_dom_post ~ JTHFT_non_dom_pre + n_pretests + is_trained + age + DASH
   Data: fingers_tap_data (Number of observations: 21)
Samples: 4 chains, each with iter = 10000; warmup = 5000; thin = 1;
         total post-warmup samples = 20000

Population-Level Effects:
                  Estimate Est.Error l-95% CI u-95% CI Rhat Bulk_ESS Tail_ESS
Intercept             0.01      0.33    -0.64     0.67 1.00    20766    15374
JTHFT_non_dom_pre     0.38      0.24    -0.09     0.85 1.00    14589    14102
n_pretests1           0.51      0.47    -0.42     1.44 1.00    17911    14947
is_trained1          -0.25      0.48    -1.22     0.70 1.00    14364    14857
age                  -0.31      0.21    -0.73     0.12 1.00    19584    13367
DASH                 -0.04      0.24    -0.51     0.43 1.00    17464    14595

Family Specific Parameters:
      Estimate Est.Error l-95% CI u-95% CI Rhat Bulk_ESS Tail_ESS
sigma     0.90      0.18     0.63     1.33 1.00    15360    14027

    Estimate Est.Error      Q2.5     Q97.5
R2 0.4311617 0.1124369 0.1699757 0.6011411

Hypothesis Tests for class b:
         Hypothesis Estimate Est.Error CI.Lower CI.Upper Evid.Ratio Post.Prob
1 (is_trained1) < 0    -0.25      0.48    -1.04     0.53       2.34      0.70
2         (age) > 0    -0.31      0.21    -0.65     0.05       0.08      0.07
```

```
3       (DASH) < 0    -0.04      0.24    -0.43    0.34       1.40      0.58
4 (n_pretests1) > 0    0.51      0.47    -0.26    1.28       6.55      0.87
```

1.4.4 JTHFT_non_dom model with class interaction

```
Family: gaussian
  Links: mu = identity; sigma = identity
Formula: JTHFT_non_dom_post ~ JTHFT_non_dom_pre + is_trained * class + age + DASH
   Data: fingers_tap_data (Number of observations: 21)
Samples: 4 chains, each with iter = 10000; warmup = 5000; thin = 1;
         total post-warmup samples = 20000

Population-Level Effects:
                    Estimate Est.Error l-95% CI u-95% CI Rhat  Bulk_ESS  Tail_ESS
Intercept              -0.03      0.41    -0.86     0.78 1.00     18738     14864
JTHFT_non_dom_pre       0.30      0.26    -0.22     0.83 1.00     15816     13866
is_trained1            -0.01      0.54    -1.07     1.08 1.00     13736     12764
classC2                 0.22      0.51    -0.77     1.25 1.00     16121     13745
classE1                -0.15      0.89    -1.94     1.59 1.00     12415     10414
classE2                -0.15      0.86    -1.89     1.52 1.00     12659     10683
age                    -0.32      0.23    -0.77     0.14 1.00     16256     13642
DASH                    0.02      0.25    -0.48     0.51 1.00     15146     13806
is_trained1:classC2     0.45      0.68    -0.87     1.85 1.00     14195     13413
is_trained1:classE1    -0.16      0.88    -1.92     1.59 1.00     12221     10212
is_trained1:classE2    -0.15      0.86    -1.89     1.57 1.00     12420     11123

Family Specific Parameters:
      Estimate Est.Error l-95% CI u-95% CI Rhat  Bulk_ESS Tail_ESS
sigma     0.93      0.20     0.64     1.40 1.00    11143    12043

    Estimate  Est.Error      Q2.5    Q97.5
R2 0.4758789 0.09977843 0.2459026 0.623936

Hypothesis Tests for class b:
                              Hypothesis Estimate Est.Error CI.Lower CI.Upper Evid.Ratio Post.Prob
Star
1                      (is_trained1) < 0    -0.01      0.54    -0.88     0.88       1.02      0.51
2 (is_trained1+is_trained1:classC2) < 0     0.45      0.63    -0.58     1.47       0.30      0.23
3 (is_trained1+is_trained1:classE1) < 0    -0.16      0.97    -1.73     1.40       1.34      0.57
4 (is_trained1+is_trained1:classE2) < 0    -0.15      0.96    -1.70     1.42       1.33      0.57
5             (is_trained1:classC2) < 0     0.45      0.68    -0.63     1.59       0.33      0.25
```

1.4.5 TMTA model without class interaction

```
 Family: gaussian
  Links: mu = identity; sigma = identity
Formula: TMTA_post ~ TMTA_pre + n_pretests + is_trained + age + DASH
   Data: fingers_tap_data (Number of observations: 21)
Samples: 4 chains, each with iter = 10000; warmup = 5000; thin = 1;
         total post-warmup samples = 20000

Population-Level Effects:
            Estimate Est.Error l-95% CI u-95% CI Rhat Bulk_ESS Tail_ESS
Intercept       0.18      0.33    -0.48     0.83 1.00    19466    14272
TMTA_pre        0.44      0.23    -0.01     0.89 1.00    16765    13192
n_pretests1    -0.04      0.47    -0.97     0.90 1.00    16356    13484
is_trained1    -0.30      0.45    -1.18     0.59 1.00    16362    13324
age             0.02      0.24    -0.46     0.49 1.00    14906    13085
DASH            0.24      0.24    -0.23     0.72 1.00    15226    13490

Family Specific Parameters:
      Estimate Est.Error l-95% CI u-95% CI Rhat Bulk_ESS Tail_ESS
sigma     0.97      0.19     0.68     1.42 1.00    12271    12483

    Estimate Est.Error      Q2.5     Q97.5
R2 0.3801217 0.1117103 0.1345271 0.558303

         Hypothesis Estimate Est.Error CI.Lower CI.Upper Evid.Ratio Post.Prob
1 (is_trained1) < 0    -0.30      0.45    -1.02     0.44       3.03      0.75
2         (age) > 0     0.02      0.24    -0.38     0.40       1.16      0.54
3        (DASH) < 0     0.24      0.24    -0.15     0.63       0.17      0.15
4 (n_pretests1) > 0    -0.04      0.47    -0.81     0.74       0.88      0.47
```

1.4.6 TMTA model with class interaction

```
 Family: gaussian
  Links: mu = identity; sigma = identity
Formula: TMTA_post ~ TMTA_pre + is_trained * class + age + DASH
   Data: fingers_tap_data (Number of observations: 21)
Samples: 4 chains, each with iter = 10000; warmup = 5000; thin = 1;
         total post-warmup samples = 20000

Population-Level Effects:
                    Estimate  Est.Error  l-95% CI  u-95% CI  Rhat  Bulk_ESS  Tail_ESS
Intercept              -0.00       0.40     -0.80      0.81  1.00     15087     15356
TMTA_pre                0.50       0.24      0.02      0.95  1.00     15658     12700
is_trained1             0.21       0.56     -0.89      1.30  1.00     10911     12681
classC2                 0.46       0.53     -0.60      1.51  1.00     12878     13834
classE1                -0.19       0.88     -1.97      1.55  1.00     14281     11679
classE2                -0.15       0.86     -1.90      1.54  1.00     13838     11377
age                     0.02       0.24     -0.46      0.49  1.00     15779     13868
DASH                    0.16       0.22     -0.28      0.61  1.00     15770     13812
is_trained1:classC2    -1.21       0.80     -2.81      0.29  1.00      9798     12089
is_trained1:classE1    -0.20       0.88     -1.98      1.50  1.00     14464     12126
is_trained1:classE2    -0.16       0.86     -1.92      1.53  1.00     14158     11543

Family Specific Parameters:
      Estimate Est.Error l-95% CI u-95% CI Rhat Bulk_ESS Tail_ESS
sigma     0.90      0.20     0.60     1.37 1.00     9775    10726

    Estimate Est.Error      Q2.5     Q97.5
R2 0.4788965 0.1077302 0.2351561 0.6461209

Hypothesis Tests for class b:
                           Hypothesis Estimate Est.Error CI.Lower CI.Upper Evid.Ratio Post.Prob
1                    (is_trained1) < 0     0.21      0.56    -0.71     1.12       0.53      0.35
2 (is_trained1+is_trained1:classC2) < 0    -1.00      0.64    -2.04     0.06      16.15      0.94
3 (is_trained1+is_trained1:classE1) < 0     0.02      0.96    -1.55     1.55       0.96      0.49
4 (is_trained1+is_trained1:classE2) < 0     0.06      0.95    -1.49     1.60       0.90      0.47
5            (is_trained1:classC2) < 0    -1.21      0.80    -2.54     0.06      15.91      0.94
```

1.4.7 TMTB model without class interaction

```
 Family: gaussian
  Links: mu = identity; sigma = identity
Formula: TMTB_post ~ TMTB_pre + n_pretests + is_trained + age + DASH
   Data: fingers_tap_data (Number of observations: 19)
Samples: 4 chains, each with iter = 10000; warmup = 5000; thin = 1;
         total post-warmup samples = 20000

Population-Level Effects:
            Estimate Est.Error l-95% CI u-95% CI Rhat Bulk_ESS Tail_ESS
Intercept      -0.33      0.37    -1.07     0.41 1.00    16560    13523
TMTB_pre        0.42      0.24    -0.05     0.89 1.00    15543    12732
n_pretests1    -0.07      0.50    -1.05     0.94 1.00    16009    13465
is_trained1     0.48      0.48    -0.49     1.42 1.00    14780    12296
age             0.07      0.24    -0.41     0.55 1.00    13929    12831
DASH            0.37      0.25    -0.13     0.86 1.00    14011    12553

Family Specific Parameters:
      Estimate Est.Error l-95% CI u-95% CI Rhat Bulk_ESS Tail_ESS
sigma     1.00      0.21     0.68     1.51 1.00    10487    12588
   Estimate Est.Error      Q2.5     Q97.5
R2 0.375349 0.1147521 0.1262941 0.5594042

Hypothesis Tests for class b:
         Hypothesis Estimate Est.Error CI.Lower CI.Upper Evid.Ratio Post.Prob
1 (is_trained1) < 0     0.48      0.48    -0.31     1.26       0.17      0.15
2         (age) > 0     0.07      0.24    -0.33     0.46       1.62      0.62
3        (DASH) < 0     0.37      0.25    -0.03     0.77       0.07      0.06
4 (n_pretests1) > 0    -0.07      0.50    -0.89     0.77       0.79      0.44
```

1.4.8 TMTB model with class interaction

```
Family: gaussian
  Links: mu = identity; sigma = identity
Formula: TMTB_post ~ TMTB_pre + is_trained * class + age + DASH
   Data: fingers_tap_data (Number of observations: 19)
Samples: 4 chains, each with iter = 10000; warmup = 5000; thin = 1;
         total post-warmup samples = 20000

Population-Level Effects:
                    Estimate Est.Error l-95% CI u-95% CI Rhat Bulk_ESS Tail_ESS
Intercept              -0.37      0.46    -1.29     0.55 1.00    18370    14807
TMTB_pre                0.42      0.26    -0.10     0.92 1.00    18503    13295
is_trained1             0.33      0.60    -0.82     1.55 1.00    16110    14301
classC2                 0.07      0.58    -1.11     1.22 1.00    17408    14319
classE1                 0.10      0.88    -1.67     1.86 1.00    15146    11840
classE2                 0.15      0.88    -1.59     1.98 1.00    16090    11936
age                     0.06      0.26    -0.46     0.59 1.00    15858    13163
DASH                    0.39      0.28    -0.16     0.94 1.00    16984    14336
is_trained1:classC2     0.12      0.76    -1.39     1.65 1.00    17021    14351
is_trained1:classE1     0.07      0.88    -1.67     1.88 1.00    15479    11740
is_trained1:classE2     0.15      0.89    -1.61     1.93 1.00    15883    12126

Family Specific Parameters:
      Estimate Est.Error l-95% CI u-95% CI Rhat Bulk_ESS Tail_ESS
sigma     1.08      0.25     0.72     1.68 1.00    10735    11193

   Estimate Est.Error      Q2.5     Q97.5
R2 0.4020413 0.1007473 0.1837086 0.5647358

Hypothesis Tests for class b:
                          Hypothesis Estimate Est.Error CI.Lower CI.Upper Evid.Ratio Post.Prob
1                     (is_trained1) < 0     0.33      0.60    -0.62     1.33       0.40      0.28
2 (is_trained1+is_trained1:classC2) < 0     0.45      0.77    -0.81     1.71       0.37      0.27
3 (is_trained1+is_trained1:classE1) < 0     0.41      0.99    -1.18     2.03       0.50      0.33
4 (is_trained1+is_trained1:classE2) < 0     0.48      0.98    -1.10     2.08       0.42      0.30
5             (is_trained1:classC2) < 0     0.12      0.76    -1.10     1.37       0.78      0.44
```

1.4.9 TMT_delta model without class interaction

```
Family: gaussian
  Links: mu = identity; sigma = identity
Formula: TMT_delta_post ~ TMT_delta_pre + n_pretests + is_trained + age + DASH
   Data: fingers_tap_data (Number of observations: 19)
Samples: 4 chains, each with iter = 10000; warmup = 5000; thin = 1;
         total post-warmup samples = 20000

Population-Level Effects:
              Estimate Est.Error l-95% CI u-95% CI Rhat Bulk_ESS Tail_ESS
Intercept        -0.35      0.38    -1.09     0.41 1.00    17732    12765
TMT_delta_pre     0.33      0.26    -0.19     0.83 1.00    15967    12529
n_pretests1      -0.15      0.51    -1.17     0.86 1.00    17224    14476
is_trained1       0.51      0.49    -0.48     1.47 1.00    15773    13247
age               0.09      0.25    -0.41     0.59 1.00    13930    12400
DASH              0.33      0.26    -0.18     0.83 1.00    14987    13025

Family Specific Parameters:
      Estimate Est.Error l-95% CI u-95% CI Rhat Bulk_ESS Tail_ESS
sigma     1.03      0.22     0.71     1.56 1.00    10855    12431

    Estimate Est.Error       Q2.5     Q97.5
R2 0.3263214 0.1108469 0.09961406 0.5172946

Hypothesis Tests for class b:
         Hypothesis Estimate Est.Error CI.Lower CI.Upper Evid.Ratio Post.Prob
1 (is_trained1) < 0     0.51      0.49    -0.30     1.32       0.17      0.15
2         (age) > 0     0.09      0.25    -0.32     0.50       1.74      0.63
3        (DASH) < 0     0.33      0.26    -0.10     0.75       0.11      0.10
4 (n_pretests1) > 0    -0.15      0.51    -0.99     0.69       0.63      0.39
```

1.4.10 TMT_delta model with class interaction

```
Family: gaussian
  Links: mu = identity; sigma = identity
Formula: TMT_delta_post ~ TMT_delta_pre + is_trained * class + age + DASH
   Data: fingers_tap_data (Number of observations: 19)
Samples: 4 chains, each with iter = 10000; warmup = 5000; thin = 1;
         total post-warmup samples = 20000

Population-Level Effects:
                    Estimate Est.Error l-95% CI u-95% CI Rhat  Bulk_ESS  Tail_ESS
Intercept              -0.37      0.47    -1.29     0.55 1.00     18933     15082
TMT_delta_pre           0.32      0.28    -0.24     0.86 1.00     17299     13014
is_trained1             0.22      0.60    -0.93     1.44 1.00     15628     13764
classC2                -0.01      0.60    -1.19     1.17 1.00     18168     13223
classE1                 0.13      0.88    -1.62     1.95 1.00     15496     12270
classE2                 0.22      0.89    -1.55     1.99 1.00     15125     12221
age                     0.09      0.28    -0.47     0.65 1.00     15454     12338
DASH                    0.36      0.28    -0.19     0.91 1.00     16362     14086
is_trained1:classC2     0.31      0.78    -1.21     1.90 1.00     15742     13735
is_trained1:classE1     0.13      0.88    -1.62     1.90 1.00     15766     12929
is_trained1:classE2     0.23      0.88    -1.50     2.04 1.00     15335     12444

Family Specific Parameters:
      Estimate Est.Error l-95% CI u-95% CI Rhat  Bulk_ESS Tail_ESS
sigma     1.10      0.25     0.73     1.68 1.00     10994    12401

    Estimate  Est.Error      Q2.5     Q97.5
R2 0.3689947 0.09858325 0.1586638 0.5329625

Hypothesis Tests for class b:
                                  Hypothesis Estimate Est.Error CI.Lower CI.Upper Evid.Ratio Post.Prob
1                     (is_trained1) < 0     0.22      0.60    -0.73     1.21       0.55      0.35
2 (is_trained1+is_trained1:classC2) < 0     0.53      0.78    -0.76     1.81       0.32      0.24
3 (is_trained1+is_trained1:classE1) < 0     0.35      1.00    -1.25     2.00       0.55      0.35
4 (is_trained1+is_trained1:classE2) < 0     0.45      0.98    -1.12     2.02       0.45      0.31
5             (is_trained1:classC2) < 0     0.31      0.78    -0.94     1.61       0.52      0.34
```

1.4.11 Polhemus model without class interaction

```
 Family: beta
  Links: mu = logit; phi = identity
Formula: Polhemus_post ~ Polhemus_pre + n_pretests + is_trained + age + DASH
   Data: fingers_tap_data (Number of observations: 20)
Samples: 4 chains, each with iter = 10000; warmup = 5000; thin = 1;
         total post-warmup samples = 20000

Population-Level Effects:
             Estimate Est.Error l-95% CI u-95% CI Rhat Bulk_ESS Tail_ESS
Intercept       -3.52      0.57    -4.60    -2.40 1.00     5674     9947
Polhemus_pre     6.20      3.48    -0.24    11.79 1.00     5746    15528
n_pretests1      0.49      0.47    -0.47     1.40 1.00    10178    11595
is_trained1     -0.15      0.44    -1.02     0.69 1.00    11124    11080
age              0.02      0.25    -0.49     0.50 1.00    12052    12455
DASH             0.07      0.21    -0.38     0.45 1.00    11176    11324

Family Specific Parameters:
    Estimate Est.Error l-95% CI u-95% CI Rhat Bulk_ESS Tail_ESS
phi    22.23     10.84     6.71    47.62 1.00     5451     9418

    Estimate Est.Error       Q2.5    Q97.5
R2 0.4761384 0.2795128 0.02983493 0.830898

Hypothesis Tests for class b:
         Hypothesis Estimate Est.Error CI.Lower CI.Upper Evid.Ratio Post.Prob
1 (is_trained1) < 0    -0.15      0.44    -0.88     0.56       1.77      0.64
2         (age) > 0     0.02      0.25    -0.40     0.42       1.15      0.53
3        (DASH) < 0     0.07      0.21    -0.30     0.39       0.53      0.35
4 (n_pretests1) > 0     0.49      0.47    -0.30     1.25       6.11      0.86
```

1.4.12 Polhemus model with class interaction

```
Family: beta
  Links: mu = logit; phi = identity
Formula: Polhemus_post ~ Polhemus_pre + is_trained * class + age + DASH
   Data: fingers_tap_data (Number of observations: 20)
Samples: 4 chains, each with iter = 10000; warmup = 5000; thin = 1;
         total post-warmup samples = 20000

Population-Level Effects:
                    Estimate Est.Error l-95% CI u-95% CI Rhat Bulk_ESS Tail_ESS
Intercept              -3.42      0.77    -4.91    -2.02 1.00     6911    12748
Polhemus_pre            6.08      3.82    -0.38    12.80 1.00     6585    13750
is_trained1             0.11      0.57    -1.07     1.22 1.00    11176    12252
classC2                -0.25      0.58    -1.41     0.88 1.00    11136    12564
classE1                -0.54      0.95    -2.55     1.26 1.00    15009    10692
classE2                -0.06      0.86    -1.76     1.67 1.00    14734    10818
age                     0.02      0.24    -0.45     0.50 1.00    15604    12378
DASH                    0.11      0.22    -0.34     0.52 1.00    13529    12234
is_trained1:classC2     0.32      0.68    -1.01     1.69 1.00    14482    13684
is_trained1:classE1    -0.54      0.95    -2.56     1.25 1.00    14887    10961
is_trained1:classE2    -0.07      0.85    -1.78     1.57 1.00    14226    10731

Family Specific Parameters:
    Estimate Est.Error l-95% CI u-95% CI Rhat Bulk_ESS Tail_ESS
phi    24.99     12.68     7.61    55.19 1.00     7353    11700

   Estimate Est.Error       Q2.5     Q97.5
R2 0.5096121 0.264892 0.06602196 0.8568316

Hypothesis Tests for class b:
                            Hypothesis Estimate Est.Error CI.Lower CI.Upper Evid.Ratio Post.Prob
1                     (is_trained1) < 0     0.11      0.57    -0.84     1.04       0.71      0.42
2 (is_trained1+is_trained1:classC2) < 0     0.43      0.61    -0.62     1.38       0.30      0.23
3 (is_trained1+is_trained1:classE1) < 0    -0.43      1.04    -2.18     1.20       1.95      0.66
4 (is_trained1+is_trained1:classE2) < 0     0.05      0.97    -1.54     1.61       0.90      0.47
5             (is_trained1:classC2) < 0     0.32      0.68    -0.77     1.45       0.46      0.32
```

1.4.13 Polhemus_del model without class interaction

```
 Family: beta
  Links: mu = logit; phi = identity
Formula: Polhemus_del_post ~ Polhemus_del_pre + n_pretests + is_trained + age + DASH + (1 |
participant)
   Data: fingers_tap_data (Number of observations: 20)
Samples: 4 chains, each with iter = 10000; warmup = 5000; thin = 1;
         total post-warmup samples = 20000

Group-Level Effects:
~participant (Number of levels: 14)
              Estimate Est.Error l-95% CI u-95% CI Rhat Bulk_ESS Tail_ESS
sd(Intercept)     0.87      0.37     0.13     1.66 1.00     2620     3268

Population-Level Effects:
                 Estimate Est.Error l-95% CI u-95% CI Rhat Bulk_ESS Tail_ESS
Intercept           -3.35      0.49    -4.34    -2.43 1.00     7087    10827
Polhemus_del_pre     2.36      2.21    -0.82     7.50 1.00     9716    10054
n_pretests1          0.45      0.52    -0.54     1.50 1.00     7590    10905
is_trained1          0.28      0.51    -0.70     1.30 1.00     9187    12478
age                 -0.17      0.34    -0.86     0.48 1.00     7983     9735
DASH                -0.08      0.21    -0.51     0.34 1.00     8462    11891

Family Specific Parameters:
    Estimate Est.Error l-95% CI u-95% CI Rhat Bulk_ESS Tail_ESS
phi    63.14     39.84    12.97   161.50 1.00     2837     6229

    Estimate Est.Error       Q2.5     Q97.5
R2 0.6933929 0.2433799 0.09076583 0.9447643

Hypothesis Tests for class b:
         Hypothesis Estimate Est.Error CI.Lower CI.Upper Evid.Ratio Post.Prob
1 (is_trained1) < 0     0.28      0.51    -0.53     1.12       0.41      0.29
```

```
2          (age) > 0    -0.17      0.34    -0.74     0.37       0.43      0.30
3         (DASH) < 0    -0.08      0.21    -0.43     0.27       1.89      0.65
4 (n_pretests1) > 0     0.45      0.52    -0.38     1.31       4.41      0.82
```

1.4.14 Polhemus_del model without class interaction

```
Family: beta
  Links: mu = logit; phi = identity
Formula: Polhemus_del_post ~ Polhemus_del_pre + is_trained * class + age + DASH
   Data: fingers_tap_data (Number of observations: 20)
Samples: 4 chains, each with iter = 10000; warmup = 5000; thin = 1;
         total post-warmup samples = 20000

Population-Level Effects:
                    Estimate Est.Error l-95% CI u-95% CI Rhat Bulk_ESS Tail_ESS
Intercept              -3.53      0.72    -5.04    -2.30 1.00     6508    10376
Polhemus_del_pre        4.57      3.37    -0.55    11.03 1.00     6844    10947
is_trained1             0.14      0.55    -0.94     1.25 1.00     9960    11862
classC2                 0.41      0.59    -0.72     1.61 1.00     8292    10883
classE1                 0.09      0.88    -1.65     1.85 1.00    13388    11457
classE2                 0.24      0.87    -1.46     2.00 1.00    13110    10970
age                    -0.19      0.24    -0.65     0.28 1.00    11807    12860
DASH                    0.13      0.20    -0.29     0.49 1.00    13000    11613
is_trained1:classC2     0.58      0.66    -0.68     1.91 1.00    11853    12556
is_trained1:classE1     0.09      0.88    -1.65     1.84 1.00    14078    11213
is_trained1:classE2     0.24      0.86    -1.46     2.00 1.00    13447    11022

Family Specific Parameters:
    Estimate Est.Error l-95% CI u-95% CI Rhat Bulk_ESS Tail_ESS
phi    26.31     13.79     8.62    60.82 1.00     7322    13050

    Estimate Est.Error      Q2.5     Q97.5
R2 0.3599727 0.2293004 0.0527204 0.8123338

Hypothesis Tests for class b:
                          Hypothesis Estimate Est.Error CI.Lower CI.Upper Evid.Ratio Post.Prob
1                  (is_trained1) < 0     0.14      0.55    -0.77     1.06       0.64      0.39
2 (is_trained1+is_trained1:classC2) < 0     0.72      0.57    -0.24     1.61       0.11      0.10
3 (is_trained1+is_trained1:classE1) < 0     0.24      0.99    -1.36     1.84       0.66      0.40
4 (is_trained1+is_trained1:classE2) < 0     0.39      0.97    -1.19     1.98       0.51      0.34
5             (is_trained1:classC2) < 0     0.58      0.66    -0.48     1.69       0.23      0.19
```

1.5 Model predictions for class x training interactions

The model's predictions for the training by class interaction are illustrated in Figures 3–5.

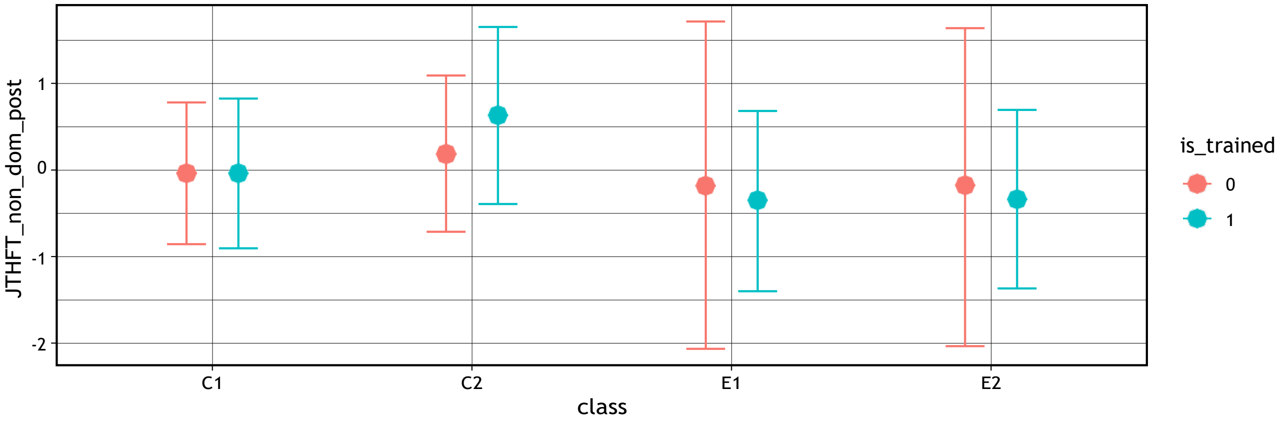

Figure 3. Fitted results (standardized) for JTHFT (non-dominant hand) post-test, grouped by class and training. A lower score is better. Due to the experimental design, there were no post-tests for

untrained participants in classes E1 and E2 – their fits shown in this figure are the model's best estimates given the remaining data.

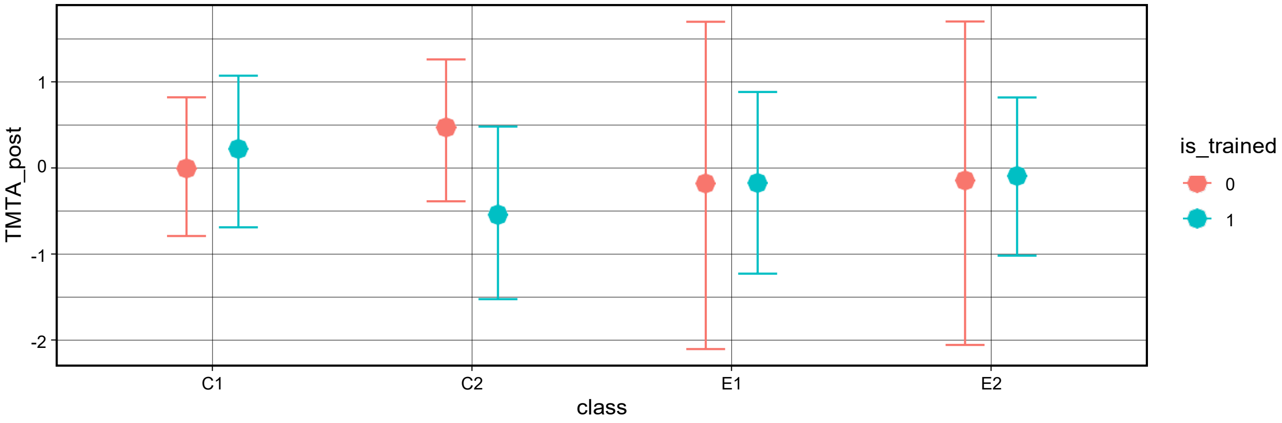

Figure 4. Fitted results (standardized) for the TMTA post-test, grouped by class and training. A lower score is better. Due to the experimental design, there were no post-tests for untrained participants in classes E1 and E2 – their fits shown in this figure are the model's best estimates given the remaining data..

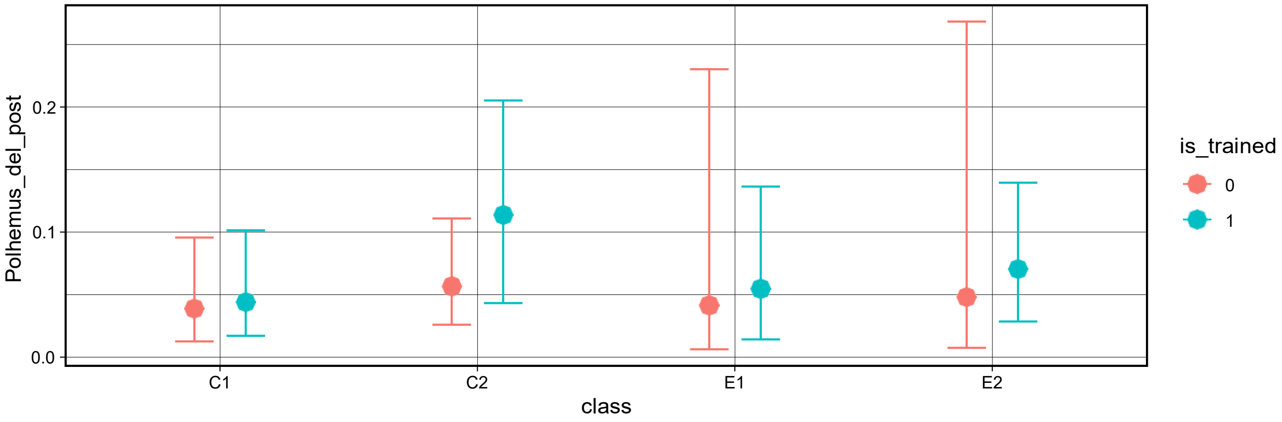

Figure 5. Fitted results (standardized) for the delayed Polhemus post-test, grouped by class and training. A lower score is better. Due to the experimental design, there were no post-tests for untrained participants in classes E1 and E2 – their fits shown in this figure are the model's best estimates given the remaining data.
